# The unspoken grief of multiple stillbirths in rural Pakistan: an interpretative phenomenological study

**DOI:** 10.1186/s12905-022-01622-3

**Published:** 2022-02-22

**Authors:** Muhammad Asim, Sehrish Karim, Hajra Khwaja, Waqas Hameed, Sarah Saleem

**Affiliations:** Department of Community Health Sciences, Aga Khan University, Stadium Road, PO Box 3500, Karachi, 74800 Pakistan

**Keywords:** Multiple stillbiths, Mental health, Spiritual treatment, Social wellbeing, Stigma, Phenomenology

## Abstract

**Background:**

Pakistan has the highest rate of stillbirth (30.6 stillbirths per 1000 total births) as compared to other South Asian countries. The psychological impact of stillbirths on bereaved women is well documented; however, there is a dearth of literature on lived experiences of women with multiple stillbirths in Pakistan.

**Objective:**

The purpose of this research is to understand the lived experiences of women who had multiple stillbirths in Thatta, Pakistan.

**Methods:**

An interpretative phenomenological study was conducted in district Thatta with eight women who experienced more than one stillbirth. A semi-structured in-depth interview guide was used for data collection. The data were analyzed by using thematic analysis approach.

**Results:**

The results of this study show that experiencing multiple stillbirths has a devastating impact on women’s mental and social wellbeing. The women who experienced multiple stillbirths are stigmatized as “child-killer” or cursed or being punished by God. They are avoided in social gatherings within the families and community, because of these social pressures these women seek spiritual and religious treatment, and struggle to conceive again to deliver a live baby. It was observed that the psycho-social and medical needs of these bereaved women remain unaddressed not only by the healthcare system but also by the society at large.

**Conclusions:**

The physical, social and mental well-being of women who experience multiple stillbirth are at stake. These women are being considered social outcast. Health care providers including physicians, lady health workers, and traditional birth attendants should be trained on provision of psychosocial support along with the routine care that they provide in communities and health facilities. The health care providers should also inform the bereaved women about the biomedical causes of stillbirths that would be helpful to mitigate the stigma associated with stillbirths. Moreover, the health care providers should also counsel family members especially in-laws of these sorrowful women about the biomedical causes of stillbirths that would also be helpful to mitigate the stigma associated with stillbirths.

## Introduction

Each year, 2.6 million babies are born without any sign of life across the globe, and almost half of these deaths occur during delivery [1]. The women who belong to low-and middle-income countries (LMIC) are twenty times more likely to deliver stillbirth as compared to women living in high-income countries [2]. Three fourth of stillbirths take place in sub-Saharan African and Asian countries [3]. Pakistan has one of the highest stillbirth rate (30.6 stillbirths per 1000 total births) in South Asian (18.2 stillbirths per 1000 total births), and across the globe (13.9 stillbirths per 1000 total births) [4]. It is estimated that 1 in 200 pregnancies results in a stillbirth in high-income countries as compared to 8.6 in 200 pregnancies in Pakistan [3, 5]. According to Global Network’s Population-Based Birth Registry, functional in Thatta the stillbirth rate is even higher (56.9 per 1000 births) [6], that is also the highest across all districts in Pakistan.

Stillbirth is a life-changing event for a woman and can have devastating mental, physical, and social impact, with negative effects on interpersonal relationships within family and community [7]. Indirect and intangible costs of stillbirth are extensive, and are usually met by families and grieving women alone [8]. The psychological effects of stillbirth adversely impact the daily functioning, relationships, and employment of women who experience stillbirths [9]. Especially the women who experience stillbirths face stigma, rejection, and abuse not only from the husband and family members but also from the community [10] and health care providers for not taking care of pregnancy [11].

Different studies from South Asia and Africa reported the risk factors and determinants of stillbirths. For example, women age < 20 years and age > 35 years are at higher risk of having a stillbirth. Moreover, compared to parity 1–2, zero parity and parity > 3 are both associated with increased stillbirth, and compared to women with any prenatal care, women with no prenatal care had a significantly increased risk of stillbirth in all sites. Women who had prolonged labour ≥ 24 h, who had uterine rupture and with lower and higher birth weight babies are more likely to deliver stillborn babies [12].

The re-occurrence of stillbirth is quite high in subsequent pregnancies in women who have had experienced a stillbirth [13–15]. Various beliefs and stigma are particularly associated with women who experience multiple stillbirths [7]. Particularly, a woman who repeatedly loses her newborns is referred to as having evil or ancestral spirit inside her body that kills the newborn. In LMICs, few studies also reported that such women are labelled as “child-killer” in the community. [10, 16]. Moreover, such women are considered unfortunate and not warmly welcomed to attend social events [16]. Furthermore, young women also avoid having social interaction with women who experienced stillbirth due to a perceived fear of contracting adverse pregnancy outcomes [11, 17, 18]. Moreover, these women are widely neglected in the family and remain under threat of being divorced or her husband may marry another woman [10, 16, 19, 20].

Bereaved women have difficulty to express how they feel after experiencing stillbirth and are unlikely to mourn publicly because stillbirth is not deemed to be a human death [10, 19, 21–23]. Stillborn babies are quickly disposed of without fuss and by informing only close relatives and other community members [16]. The family rarely shows the body of the stillborn to the mother, to extended family, and the community due to the stigma attached to stillbirths [11]. Furthermore, people are hesitant to talk about stillbirths fearing such action could evoke future malice or lead to infertility [23] and other adverse events [10]. Moreover, people also hide stillbirth due to false beliefs such as effect of an the evil eye, the influence of the evil spirit, and black magic [23].

In Pakistan fertility and childbearing are considered a highly prized and blessing event for women [24], and women who deliver stillborn are considered wretched and stigmatized. Women who experience multiple stillbirths (more than one stillbirth) face social stigma, depression, and social isolation in society. In such situation different myths and taboos revolve around women who experience multiple stillbirths. To the best of our knowledge, the previous studies conducted in Pakistan primarily focused on the rate, determinants, and causes of stillbirths [6, 25, 26]. However, understanding the lived experiences of multiple stillbirths by women is not well documented in Pakistan. The objective of this study is to understand the lived experiences of women who encountered multiple stillbirths in the rural district of Thatta, Pakistan.

## Methods

### Study design

The qualitative methodology was employed to understand and capture the meanings, experiences, grief, and stigma associated with stillbirths. For this purpose, the phenomenological research design was chosen to carry out this research. Phenomenology is a well-established qualitative research approach that is widely used in health psychology to examine an individual's lived experiences with a certain phenomenon [27]. Grief, stigma, and pain are prime exemplars of such phenomena to understand how people make sense of their experiences in their daily lives [28]. The objective and essence of the interpretative phenomenological analysis approach are to enable the environment to explore the lived experience of the participants and to describe the research findings through their experiences and bracketing researcher’s own biases and experiences. The research team neither experienced stillbirths nor have a close association with women who experienced stillbirths.

### Study setting

The study was conducted in rural villages of district Thatta Sindh, Pakistan. Thatta is predominantly a rural district situated in a coastal area in the southern part of Pakistan. Thatta is categorized in the low human development index in Pakistan [29] where only 17% of women are literate as compared to 47% across Pakistan [30]. The population of District Thatta is one million people and 90% of people live in rural areas. Women are only engaged in the informal economy such as working in agriculture fields and in informal labor [31]. We chose Thatta for this study because of reported highest stillbirth rate (43.2 per 1000 total births) across Pakistan [6].

### Study participants

We selected and interviewed only those women who had experienced two or more stillbirths, with last stillbirth occurring within the period of last 12 months from the date of interview i.e. June 2018–May 2019. These women were identified by the key informants who provide healthcare services to women and are also part of these communities such as lady health workers (LHWs), traditional birth attendants (TBAs), quacks (unregistered health practitioners), local political leaders, and religious leaders. These key informants hold important position in the community and villages and have direct and in-direct contact with bereaved women or with their families. They identified the women who experienced multiple stillbirths and also helped us to reach out these women for conducting interviews. We kept in contact with key informants from June to July 2019 to get to know about study participants.

### Eligibility criteria

The key informants identified 22 women who experienced more than two stillbirths. Our eligibility criteria to recruit the women who experienced multiple stillbirths (more than one) and experienced the last stillbirth within the last 12 months. Moreover, we also excluded the 05 women who did not give the consent to participate in the study. Only 8 women met our eligibility criteria.

### Interview guide

A semi-structured interview guide was developed by MA after reviewing literature and discussing it with psychologists and health care providers. The interview guide was pilot tested on two women who experienced stillbirths from an adjacent district. The interview guide was modified after pilot testing and during the actual data collection process as we learned more about social stigma and local cultural taboos associated with the stillbirths. The major themes included in the semi-structured guide were meaning and symbols attached with stillbirths, the social and psychological impact of stillbirth on women, social support, and coping strategies adopted by the affected women. The semi-structured interview guide had some broad questions such as: emotional and psychological experiences after stillbirth, what types of challenges faced after stillbirth, social support mechanism, health-seeking behavior, and socio-cultural issues of bereaved women.

### Data collection process

Data were collected from June to July 2019. In total, we conducted eight in-depth interviews with eligible women (see Table 1). We conducted interviews at women’s homes and at the time of the interview only the research team and interviewee were present at a separate place. A qualitative trained social science male graduate conducted face to face interview along with a female note-taker. The researchers introduced themselves as physicians and researchers from the Aga Khan University Karachi who is interested to interact with women who experienced multiple stillbirths to get to know about their lives. In-depth interviews lasted between 30 and 40 min. The interviews were recorded through a digital audio recorder and note-taking after the verbal consent of the participants. All study participants were illiterate due to a lack of formal education, so verbal consent was taken from the study participants. After each interview, a debriefing session was held with research teams to contextualize the information and interpretations of the key terms. Detailed field notes were prepared during the process of data collection and were used as a reference for interviews to note down the expressions of participants and outlining the initial codes of the provided data.

**Table 1:** Socio-demographic characteristics of interviewed women (n = 8)

	Age of women in a range	Age at marriage	Education status	No. of stillbirths	No. of living children	Miscarriages/Abortion	Gestation at time of loss	Place of loss of a child	Time since the loss (months) at interview
1	35–39	14	No education	5	3	3	09th months	BHU	2
2	26–30	16	No education	2	0	0	07th months	One the way	5
3	15–19	13	No education	2	0	2	08th months	Home	8
4	35–39	17	No education	2	0	0	08th months	Home	11
5	35–39	20	No education	2	0	0	08th months	On the way	4
6	15–19	17	No education	2	1	0	09th month	BHU	5
7	35–39	14	No education	6	0	3	09th month	DHQ	3
8	25–29	16	No education	2	0	0	08th month	BHU	2

### Data analysis

The data were analysed through the interpretative phenomenological analysis approach (IPA). This analysis approach is widely used in psychological qualitative research that explores personal lived experiences related to complex, ambiguous, and emotionally laden areas [32]. Neutrality is the core component of phenomenology which can be attained by bracketing out the researcher’s own experiences from the phenomenon understudied [33]. Moreover, the research team tried their level best to keep apart from their learned experience through literature from the study findings. This approach focuses on lived experiences by the people who faced the situation rather than prescribed by pre-existing theoretical preconceptions.

The main objective of using IPA was to understand bereaved women’s psychological world due to experiencing multiple stillbirths. The process of data analysis was initiated during the data collection. Since we analyzed and collected data simultaneously and concluded further data collection upon reaching information saturation. The data saturation point was discussed with all co-authors during the data collection process. When researchers observed that no further new information is emerging from subsequent interviews. The further data collection was concluded with the mutual decision of all co-authors. Two co-authors (SK, HK) translated data verbatim from Urdu to English. The data were analysed through the inductive method that facilitated the emergence and identify themes from the data. Data were analysed through an interactive process by following multiple steps such as reading and re-reading the transcripts, initial noting, developing emergent themes, searching for connections across emergent themes, and looking for patterns and alternative explanations [34]. The initial coding and themes were identified by the two authors (MA, WH) after detailed reading of each transcript and field notes. A list of preliminary themes which emerged from the transcripts was discussed by all co-authors and discrepancies on over-reaching themes were resolved mutually by checked back to the transcripts. Credibility checks were achieved through triangulation with senior members of the research team (MA, and SS).

### Ethical consideration

Ethical approval was taken from the Ethics review committee of Aga Khan University, Karachi. [AKU-ERC-2020-0479-8902]. All methods were performed in accordance with the guidelines and regulations of bioethics guideline of the Aga Khan University Karachi. Moreover, a formal permission letter was obtained from the District Health Government, Thatta for field activities.Since all our study participants were illiterate, the consesnt form was read to the participants in local language, by a family member who was literate and trustworthy to the participant. Written informed consent was obtained from legally authorized representatives (LARs) literate family members of the participating women. The LARs read the written consent form to the study participant and provided the opportunity for further clarifications. The study participant understood the content of the consent form and gave consent to volunteer participation in the study before starting the interview. This study was part of the Rural Health Program of the Department of Community Health Sciences.

## Results

**Fig. 1:**
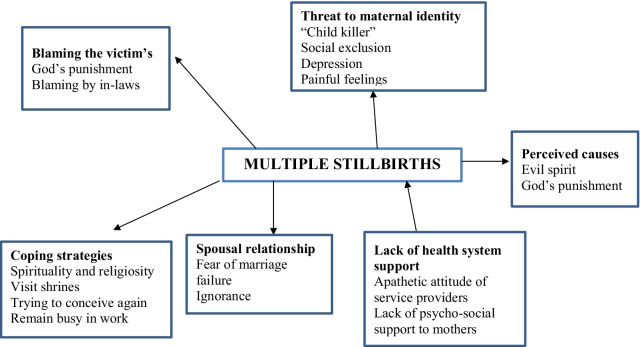
Conceptual framework of unspoken grief of multiple stillbirths

Eight women who experienced more than one stillbirth were interviewed by using a semi-structured interview guide. Seven out of eight women got married before the age of 18 years and the mean age of marriage was 16.5 years. All women who were interviewed had never been to school. Two out of eight women had more than two stillbirths and three women had also experienced miscarriages and abortions. Two out of eight women had living children. Four women experienced stillbirths in the previous 09 months, and three women experience stillbirths within last 3 months at the time of interview. Based on our analysis, the findings are presented under seven major themes that are: blaming the victims, a threat to maternal identity, spousal relationship, perceived causes, stigma, coping strategies, and lack of health system support. A conceptual framework of study themes are presented in Fig. 1.

### Blaming the victims

Stillbirths are considered an impulsive action of women and they are considered solely responsible for such incidents. Women in their interviews mentioned that stillbirths are considered as a punishment by the God to the women for being disobedient. However, if a woman experience more than one stillbirth, then she has to face more intense stress, stigma, and guilt from both household and society. For example, a woman shared that how she was blamed after experiencing a second stillbirth:

“In my first stillbirth, my husband and in-laws were wondering that selection of public health facility may be the cause of stillbirths. However, when I experienced another stillbirth then all family members started blaming me for delivering stillbirths.” (Participant, 3)

Most families in rural areas live in joint families where usually paternal grandmothers are considered as the household head. In a joint family system, women have to face the criticism of their in-laws in case of any adverse pregnancy outcome. For example, a woman who recently experienced stillbirth reported that her husband and in-laws constantly blamed on her for delivering a dead baby.

“How can I be responsible for delivering stillbirth after taking care of my pregnancy [fetus] for several months…? It is my utmost desire to have an alive baby and I cannot even think to harm my baby [fetus].” (Participant, 4)

Women during discussions shared that experiencing multiple stillbirths are considered as being possessed by witchcraft and hag which kill the fetus inside woman’s body. Such women are usually avoided for social and physical contact, and remain socially isolated in the community. It is also important to mention that older women who experienced several stillbirths and those whose babies die after livebirth pass through this painful attitude of community and are blamed as the main reason for child’s death. Moreover, women who do not have any living child also experience guilt and criticism of not taking care of themselves during conception. An older woman who experienced six stillbirths and did not have a live child voiced:

“My relative called me “child-killer” and women usually forbid their children to visit my home because I am considered as untouchable that make me feel guilty and very bad”. (Participant, 7)

### Perceived spiritual causes of stillbirths

It was noted that women who experienced multiple stillbirths believe in religious and spiritual causes of stillbirths such as having an evil spirit in the body, not following the instructions of spiritual healers, and could not visit the shrines. Experiencing multiple stillbirths put these women in such a condition where women as well as community members associate stillbirths with spiritual interventions. Such beliefs enable bereaved women to cope better with the devastating effects of experiencing stillbirths from faith healers. For example, a woman reported that:

“The reason for delivering dead babies [stillborn] is that I am possessed by an evil spirit (Jin) when I had gone to the holy shrine, a religious man said the same thing and then I believed that because of evil spirit (Jin) my babies are born dead.” (Participant, 7)

Another woman mentioned that she used to visit the holy shrine every week. However, when she could not attend the shrine and stopped the holy practices that became the major cause of stillbirth.

“After the ultrasound in the 7th month, the doctor said everything is fine. Then I stopped visiting the shrine and also stopped drinking the holy water. Due to which the evil spirit came back and my child was born dead again before the due date.” (Participant, 8)

Particularly women who experience adverse pregnancy outcomes usually seek antenatal and postnatal care from spiritual healers. Our interview reported that women had the belief that stillbirths are an outcome of evil spirits that kill the fetus and spiritual healing can cure this disease. For example, a woman shared that:

“Whole day I work in fields and in the afternoon we sleep under trees due to which most women get possessed by an evil spirit (Jin) because of which dead children are born.” (Participant, 4)

“Life and death are in God’s hands. I don’t know why my babies are born dead. A saint said that because of evil spirits (Jin) they are born dead. Then I started wearing a sacred thread, but it didn’t work and one more baby was born dead.” (Participant, 2)

Women who experienced stillbirths and do not have living children had a strong belief on supernatural powers that kill their children. Moreover, such women usually seek treatment from religious and spiritual leaders to deliver live children.

### Threat to maternal identity and stigma

The interviews reported that women who experienced multiple stillbirths had been facing maternal identity crises due to not being able to deliver a livebirth. Experiencing multiple stillbirths is associated with disability and women and family members usually try to hide stillbirths. Experiencing a stillbirth is usually associated with women’s fate and those women who are not able to have children they face a maternal identity crisis. Women professed their incompetence to give birth to a live child as a failure of their womanhood. A woman reported that:

“I felt remorse for delivering stillbirths. I felt valueless that I am not even able to deliver a live child. I think my husband will leave me because I will not be able to become a mother.” (Participant, 7)

Our study has highlighted that even women in older age who do not have children also feel incomplete and less empowered. Women who have children are considered empowered and for those who experience stillbirths, their womanhood remains questionable. According to a woman who was 39 years old and had two stillbirths reported that:

“In our society, only women who have children get respect in family and society. I feel incomplete when I think that I am not able to become a mother of any child.” (Participant, 5)

These women are marked as a disgrace to the society and are avoided in social gatherings especially those related to celebrations of marriage or childbirth, as their presence could bring bad luck to the newlyweds or to the newborn and to the mother of the newborn. That is why they are either avoided or not welcomed by people, which makes them more isolated and depressed. For example, women who experienced a stillbirth in the last 3 months voiced that:

“It is very painful for me that I cannot go to any parties. Especially when a new child is born in some house, women there, during pregnancy and in postpartum don’t meet me due to fear of losing their child.” (Participant, 7)

“Pregnant women avoid seeing and meeting me due to my disability. And no one calls me in any occasions to attend as they say that I am possessed by an evil spirit (Jin).” (Participant, 3)

### Spousal relationship

Women reported mixed responses regarding their husband’s reaction to a stillbirth. The interviewed women generally had bitter relationship with their husbands and in-laws and only a few were living with the fact considering it to be God’s will. Younger women had a better relationship with their husbands as compared to women who were in the older age group. Similarly, women who had living children were also reported to have better spousal relationship as compared to women who did not have living children. Moreover, the spousal relationship was also dependent upon the number of adverse pregnancy outcomes a woman had. For example, a 23-year old woman pointed out that:

“My husband is a simple man. He never talked about anything that hurts me. He is happy in God’s will and has never blamed me for children's deaths. We both husband and wife have been living a happy life. But I am worried about him [husband] that he might get married if I would not be able to give a livebirth.” (Participant, 8)

Women in older age groups and without children were more concerned about failing marriages and unhappy relationships. Most of the interviewed women showed their concern that their husbands will have a second wife for want of children. A 37-year-old woman explained:

“Our relationship is no more warm due to experiencing stillbirths. I cannot explain how these incidents have changed our relationship. He [husband] is no more taking interest in me”. (Participant, 7)

Our interviews revealed that women after passing the recent trauma of stillbirth are not treated well by their husbands and in laws; they are avoided and mistreated by their husbands and in-laws. They feel threatened that their marital relationship will also be affected negatively and brings a feeling of insecurity in grieving women. A woman who experienced a stillbirth 4 months back alluded to this point:

“Whenever a dead child is born my husband and in-laws would say that I am careless and my husband would stop talking to me for days. I think he is already married again or will marry soon.” (Participant, 5)

### Coping strategies

Our interviews also mentioned that women who suffered from stillbirths had different survival mechanisms to deal with society and to deal with the humiliation and misery they face. Such women keep themselves busy in household chores, visiting shrines, and remain busy with children. Particularly older women with more than two stillbirths shared that they keep themselves busy in different household chores and economic activities. For example, a 35 year old woman said:

“I keep myself busy the whole day in household chores and working at fields. I don’t keep myself free so I cannot think about my losses and get sad.” (Participant, 4)

Similarly, women usually take care of the children of their brothers-in-law in a joint family to keep themselves busy. It is important to mention that women who were married within family (cousin marriages) also seek family support such as being a caretaker of one of their children as a coping strategies to overcome their grief. Women usually receive social support from family members but stigma associated with not able to have a living children remain dominant.

“I spend my whole day with my sister’s children. I don’t go out as I don’t like people to talk about me. All of my time is spent with children.” (Participant, 7)

Some women also get some solace by visiting shrines and reflected that visiting Holy places and praying make them feel comfortable and helped them manage the pain of child loss. Moreover, young women struggle to conceive again to cope with the stress. According to a young mother who recently experienced stillbirth:

“To deal with my grief I pray and go to Holy Shrine. Going to a shrine makes me relaxed because lots of females go there whose babies were born dead or they are sad. Then I get patience by looking at them and try to conceive again.” (Participant, 6)

Myths, cultural and family pressures for being possessed by an evil eye on these women as mentioned earlier, they are not invited to any social activities. These practices forced them to adopt an alternative survival mechanism. The women resume their household duties to keep themselves busy, other than that, they also increase their engagement in spiritual and religious activities (prayer/namaz/rituals) and taking care of other’s children.

“I go everywhere for treatment whoever tells me. I have got treated with an amulet, holy water, and doctor so that my child is born alive but every time around 7–8 months my baby is born dead.” (Participant, 5)

### Lack of health system support

Interviews reported that health system support was absent to meet the needs of such women who experienced stillbirths. Participants reported that health care providers do not listen and treat properly when they visit health care facilities to let them know the cause of stillbirth. It is important to mention that health care providers, particularly physicians rarely listen to poor women who belong to rural areas due to their socio-economic status. However, women trust other types of healthcare providers such as: LHWs, TBA, and quacks that belong to the same community. Though, such providers refer women to secondary-level facilities for advanced treatment. According to a woman:

“I always ask from doctor to tell me the reason for my stillbirth and miscarriages. I want to know if I can deliver a live baby? She [the doctor] usually says, pray to God for a live baby” (Participant, 7).

Women also reported that healthcare providers only prescribe medicine without listening to their past experiences. Furthermore, healthcare providers never acknowledged their grief or recognize the traumatic impact of having a stillbirth. Moreover, women who experienced stillbirths expressed their desire to receive special care, treatment and psychological support from the physicians which they don’t get support. A woman said:

“I was just informed by the nurse that you delivered a dead baby and asked to leave the hospital because I was fine after delivery. I was shocked and nobody from the hospital was ready to tell me the cause of stillbirth. Whenever I visit a doctor, I try to share my previous delivery experiences, but the doctor never listens to me properly.” (Participant, 1).

The compromised health system response may be attributed to several factors. For example, women requiring specialized care may not be offered adequate clinical care due to the unavailability of services within rural district. Also, women’s inability to access such services in cities (tertiary care hospital) due to prohibitive cost. In terms of interpersonal care, service providers tend to ignore their information seeking by simply recommending them to pray for a live birth, mainly because of their low literacy and poor socio-economic status.

## Discussion

This is the first study that explored in-depth lived experiences of women who encountered multiple stillbirths in rural settings of Pakistan. Interviews reported that experiencing multiple stillbirths puts women in a ruinous position in society. There are different societal myths and misconceptions associated with stillbirths. Stillbirths are considered taboo in Pakistan; however, experiencing multiple stillbirths isolates women in the household and the community. Our interviews reported that women who experienced multiple stillbirths and do not have living children are blamed and considered under influence of witchcraft that kills the fetus. However, mothers who had living children did not face such stigma and labeling. Different studies from South Asia also reported that women who experience stillbirths face stigma, social isolation, and being blamed for having stillbirths [6]. Furthermore, such women are avoided having physical and social contact due to myths and taboos associated with women who experienced such incident. It is common to believe that women who experience multiple stillbirths are avoided by young pregnant women and in ceremonies related to childbirth and marriages [7]. Women avoid having social contact with women who experienced stillbirths due to various societal myths associated with stillbirths.

Our interviews noted that women who experience multiple stillbirths are considered to have an evil spirit in their body that can be transmitted to another pregnant woman. Such rumors socially isolate and stigmatize women who had experienced stillbirths. The major cause of stillbirth is considered a spiritual state, and only women can get a cure by attending spiritual healers and visiting shrines. Moreover, such women are considered to have the evil spirit in the body that kills the child. For this purpose, women who did not have living children do prefer seeking spiritual treatment by visiting shrines or religious healers. Religious and spiritual beliefs enable bereaved women to cope better with the devastating effects of the loss of a baby. Different studies also found that women who experienced stillbirths seek spiritual treatment from shrines, spiritual healers[6].

Motherhood is considered a major goal of life for many women, and if someone fails to achieve this, there is a sense of shame that this life goal is not successfully attained [35]. Our interviews reported that women who did not have children feel valueless, incomplete and feel insecure about their marital relations in a patriarchal society. Such women reported that their husband marry another woman for having children. However, women, who had living children, did not share such concern about their spouse. Different studies also pointed out that pregnancy loss negative impacts on spousal relations [36] and couples' risk of break-up is also higher after experiencing pregnancy loss [37, 38]. A phenomenological study on Israeli women also found that the loss of the pregnancy undermines the women's basic belief in their fertility and as a result threatens their meaning and role as women [39]. Such women feel under pressure to prove their capabilities to reproduce as early as possible to meet a sustainable relationship with their spouse [39]. Besides, our study also reported that bereaved women are being neglected and mistreated by their husbands and in-laws and ignore the biomedical causes of stillbirths. Particularly women who had no living children and experienced multiple stillbirths believe in the supernatural interventions that cause stillbirths. In such a situation, women face discrimination and threat to maternal identity at the household level as well as community level.

Through our study responses, we were able to assess that the victims and their families had believed an inconsiderable causes such as: influence of demon (*Jin*), and this suffering is the return of karma due to being disobedient to some spiritual saints. A study from Pakistan also highlighted that how community members associate the causes of stillbirths with religious and cultural contexts [16]. Such women are being stigmatized by society due to the stillbirth—that is considered as misfortunate. Moreover, such women and their families had both positive and negative coping strategies. It was found that the women socially isolate themselves after having a stillbirth for a certain period of time to protect themselves from the aggravation of their grief. They also immerse themselves in their household work to cope with the pain and grief of stillbirth. In our study, the women seemed to use this coping strategy to overcome their grief. A previous study highlighted the gender difference in coping styles following the loss of a baby to stillbirth. The study showed that men during suffering use the support of friends and relatives and ignore the issue; whereas, women seek spiritual and religious support, used wishful thinking about the future, and to seek support from others who suffered similar grief [6]. The difference in the grieving pattern of men and women is interesting to follow; men do not feel threatened for breaking up of the marital relationship or being blamed for stillbirth where as women constantly live with this fear and seek refuge in visiting shrines and seeking spiritual healing.

Our study revealed that spiritual healing from religious leaders, visiting shrines, and struggle to conceive again as soon as possible were the major coping mechanism of bereaved women. Religious and spiritual beliefs play a vital role particularly in the lives of bereaved women who have recently experienced stillbirth or did not have living children. Religion plays an important role in bereaved women's life for coping with the pregnancy loss that provides hope and courage [40]. Moreover, various studies also reported that most women show an overwhelming urge to become pregnant as soon as possible after perinatal death as a coping mechanism for delivering a live baby [7, 41, 42]. Becoming pregnant and having another child too soon after a perinatal loss results in unresolved grief issues [41].

Women reported that they are interested in knowing the causes of stillbirths, but physicians never listened properly. It was also noted that there is a lack of health system and social welfare support to counsel women after pregnancy loss. Moreover, there was a strong sense of dissatisfaction with the health system, and health services that are being provided by health care providers to bereaved women. Various studies from rural areas of Pakistan also reported that inaccessible healthcare facilities, poor attitude of health care providers, and poor quality of care are common problems that many bereaved women experience after stillbirths [16, 30, 43]. Lack of health system support to satisfy the healthcare needs of bereaved women may be the major reasons to seek spiritual treatment in rural areas. Moreover, women trust local healthcare providers such as: LHWs, TBAs, quacks who provide primary care and accompany them to health facilities and refer them to appropriate health facilities in case of any obstetric emergency. Similarly, different studies from rural areas of Pakistan also highlight that local TBAs and LHWs enjoy greater community acceptance and provide greater patient satisfaction. The TBAs and LHWs remain the most available and accessible health resource in most rural settings [44, 45]. Various studies also point out a lack of trust in the health care services provided by public sector hospitals in Pakistan [30, 46, 47].

### Strengths and limitations of the study

This qualitative study uncovered, and understands the multifaceted experiences of bereaved women who experienced multiple stillbirths in rural district Thatta, Pakistan. The current phenomenological study enabled us to understand the stillbirth experiences of bereaved women and their meaning to them. Moreover, this study was conducted by a team consisting of both male and female researchers to better contextualize the lived experiences of women who experienced stillbirths. The interviews were conducted by a male member (PI) along with a female research assistant. Since this study was conducted with only eight women from only one purposefully selected district of Thatta, Sindh, our results may not reflect the experiences of bereaved women residing in other geographic areas within Pakistan. Moreover, we only interviewed women who experienced the most recent stillbirth in the last 12 months and some important information may be lost for not including many women who also experienced stillbirths. We suggest some additional investigations across Pakistan designed to identify regional and cross-cultural experiences of bereaved women to improve mental health and needed social support from the health system and health care providers.

## Conclusion

Stillbirths are the major cause of psycho-social morbidity that has a long-lasting impact on women’s lives. Different cultural believes and stigmas are associated with stillbirths that isolate bereaved women in the community. Such stigmas associated with stillbirths are needed to be addressed by health care providers through provision of information on biomedical causes of stillbirths during antenatal or postnatal visits. Moreover, the health system should not only to account for and prevent stillbirths but also screen such women who experienced stillbirths to follow up and provide psycho-social and emotional support to bereaved women after experiencing stillbirths. Family support in such a crucial time can also play a significant role in coping stress of bereaved women. It is also suggested that health care providers including physicians, lady health workers, and traditional birth attendants should provide psychosocial support and inform the bereaved women about the biomedical causes of stillbirths that would be helpful to mitigate the stigma associated with stillbirths. Moreover, it is also suggested that training of maternity staff on psychosocial support of patients should be organized to strengthen the capacity of service providers. A study from rural areas of Pakistan highlights the importance of psychological intervention delivered by community-based primary health workers, and it has the potential to be integrated into health systems in resource-poor settings to address the perinatal depression [48]. Another study developed a framework of ‘doing no harm and how to put things right’ through the training of maternity staff in evidence-based bereavement care [49]. Such frameworks can be adopted and modified according to the local socio-cultural needs for the training of community based healthcare workers to address the mental health issues of bereaved women in Pakistan.

## Data Availability

The datasets used and/or analyzed during the current study are available from the corresponding author on reasonable request.
